# Sigma-1 Receptor Enhances Neurite Elongation of Cerebellar Granule Neurons via TrkB Signaling

**DOI:** 10.1371/journal.pone.0075760

**Published:** 2013-10-08

**Authors:** Yuriko Kimura, Yuki Fujita, Kumi Shibata, Megumi Mori, Toshihide Yamashita

**Affiliations:** 1 Department of Molecular Neuroscience, Graduate School of Medicine, Osaka University, Suita, Osaka, Japan; 2 JST, CREST, 5, Sanbancho, Chiyoda-ku, Tokyo, Japan; University of Louisville, United States of America

## Abstract

Sigma-1 receptor (Sig-1R) is an integral membrane protein predominantly expressed in the endoplasmic reticulum. Sig-1R demonstrates a high affinity to various synthetic compounds including well-known psychotherapeutic drugs in the central nervous system (CNS). For that, it is considered as an alternative target for psychotherapeutic drugs. On the cellular level, when Sig-1R is activated, it is known to play a role in neuroprotection and neurite elongation. These effects are suggested to be mediated by its ligand-operated molecular chaperone activity, and/or upregulation of various Ca^2+^ signaling. In addition, recent studies show that Sig-1R activation induces neurite outgrowth via neurotrophin signaling. Here, we tested the hypothesis that Sig-1R activation promotes neurite elongation through activation of tropomyosin receptor kinase (Trk), a family of neurotrophin receptors. We found that 2-(4-morpholinethyl)1-phenylcyclohexanecarboxylate (PRE-084), a selective Sig-1R agonist, significantly promoted neurite outgrowth, and K252a, a Trk inhibitor, attenuated Sig-1R-mediated neurite elongation in cerebellar granule neurons (CGNs). Moreover, we revealed that Sig-1R interacts with TrkB, and PRE-084 treatment enhances phosphorylation of Y515, but not Y706. Thus, our results indicate that Sig-1R activation promotes neurite outgrowth in CGNs through Y515 phosphorylation of TrkB.

## Introduction

Sigma-1 receptor (Sig-1R) is a brain-enriched transmembrane protein that is predominantly expressed in the endoplasmic reticulum (ER) in the subdomain close to the mitochondria known as the mitochondria-associated ER membrane [1], [2]. Sig-1R was originally identified as an opioid receptor [3], but accumulating evidences identified it as a novel ligand-operated molecular chaperone [4]. Sig-1R is expressed in various type of organs, with Sig-1R mRNA expression detected in the brain, liver, kidney, and heart of adult mice [5]. Among these organs, Sig-1R mRNA expression is highest in the brain [5]. In addition, Sig-1Rs found within the brain are well known for their high affinity to a wide variety of synthetic compounds, which are primarily psychotherapeutic drugs [6]–[10]. Sig-1R is therefore thought to contribute to drug efficacy, and is now considered an alternative target for pharmacological treatment [6]–[9]. For these reasons, Sig-1R function within the central nervous system (CNS) have become one of the major focuses of research interest of these days.

Sig-1R can promote both neuronal survival and neurite elongation [11]. However, detailed mechanisms of neurite outgrowth mediated by Sig-1R remain unknown. One possible mechanism includes tropomyosin receptor kinase (Trk) receptor. Neurotrophin binds to Trk receptor and that activates the receptor through dimerization and autophosphorylation on tyrosine residues. Once the Trk is activated, it promotes neurite outgrowth, cellular survival, and synaptic plasticity [12]–[14]. Moreover, recently, Sig-1R activation is reported to induce neurite outgrowth via neurotrophin signaling [15]. Based on these previous findings, we hypothesize that Sig-1R enhances neurite outgrowth by regulating Trk activation. Here, we demonstrated that treatment of 2-(4-morpholinethyl)1-phenylcyclohexanecarboxylate (PRE-084), a selective Sig-1R agonist, promotes neurite outgrowth in cerebellar granule neurons (CGNs) by enhancing tyrosine phosphorylation on Trk, specifically one of its family, tropomyosin receptor kinase B (TrkB).

## Results

### Sig-1R Activation Promotes CGN Neurite Elongation

**Figure 1:**
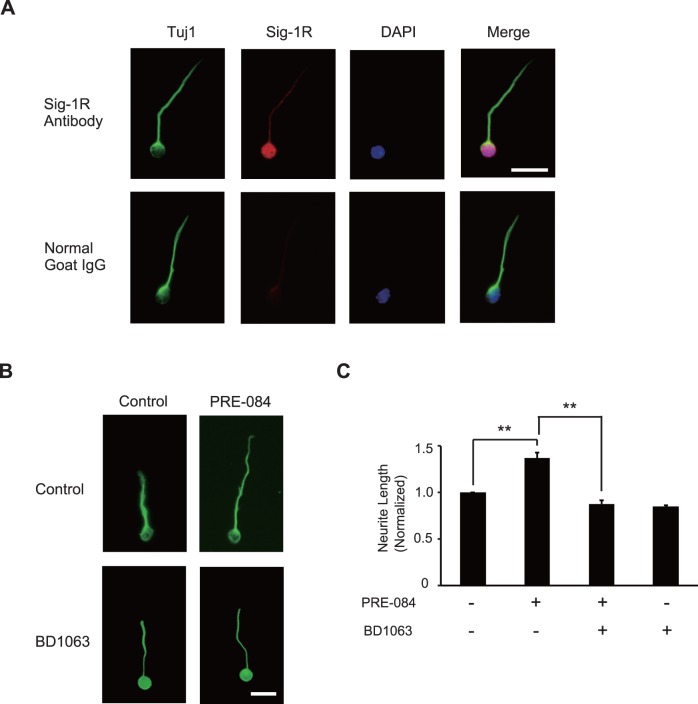
Sig-1R promotes neurite outgrowth in CGNs. (A) Immunostaining of cerebellar granule neurons (CGNs) with neuron-specific class III beta-tubulin (Tuj1) (green) and Sigma-1 receptor (Sig-1R) (red) antibodies and 4′,6′-diamidino-2-phenylindole (DAPI) (blue). Sig-1R expression was observed in the soma of Tuj1-positive neurons, but with weak expression in neurites. Only the second antibody was added to the control to eliminate the possibility of nonspecific binding. Scale bar: 20 µm. (B and C) CGNs were treated with 2-(4-morpholinethyl)1-phenylcyclohesanecarboxylate (PRE-084), a Sig-1R selective agonist, and/or 1-[3,4-dichlorophenyl]ethyl]-4-methylpiprazine (BD1063), a Sig-1R antagonist, and cultured for 24 h. Cells were then immunostained with anti-Tuj1 antibody. The representative images of CGNs are shown (B). The mean lengths of the longest neurite per neuron are represented in the graph (C). The treatment with PRE-084, significantly promoted outgrowth in the CGNs. The effect was abrogated by BD 1063. Scale bar: 20 µm, n = 3, **P<0.01, Scheffe’s test.

To examine the roles of Sig-1R on neurite outgrowth, we first examined its expression in CGNs. Cells prepared from 7- to 9-day-old C57BL/6J mice were cultured for 24 h and stained with antibodies for Sig-1R and neuron-specific class III beta-tubulin (Tuj1). We found that Sig-1R was expressed in the soma and weakly in neurites of Tuj1-positive cells (Fig. 1A). Next, we investigated the effect of Sig-1R activation on neurite outgrowth in CGNs. The cells were cultured for 24 h in either the presence or absence of PRE-084, a Sig-1R agonist, and then immunostained with anti-Tuj1 antibody. Following this treatment, neurite lengths were measured and compared between the two groups. We observed that the neurite lengths of the CGNs treated with PRE-084 were significantly increased by 37% (±6%) compared with those of the control cells. This effect was abrogated when the cells were cultured with both (1-[2-(3,4-dichlorophenyl)ethyl]-4-methylpiperazine (BD 1063), a Sig-1R antagonist, and PRE-084 (Fig. 1B and C). The treatment with BD 1063 alone showed a slight, but insignificant decrease in neurite length when compared to those of the control cells (Fig. 1B and C). These results provide strong evidence that activated Sig-1R promotes neurite outgrowth in CGNs.

### Sig-1R Activation Promotes Neurite Elongation Through the TrkB Receptor

**Figure 2:**
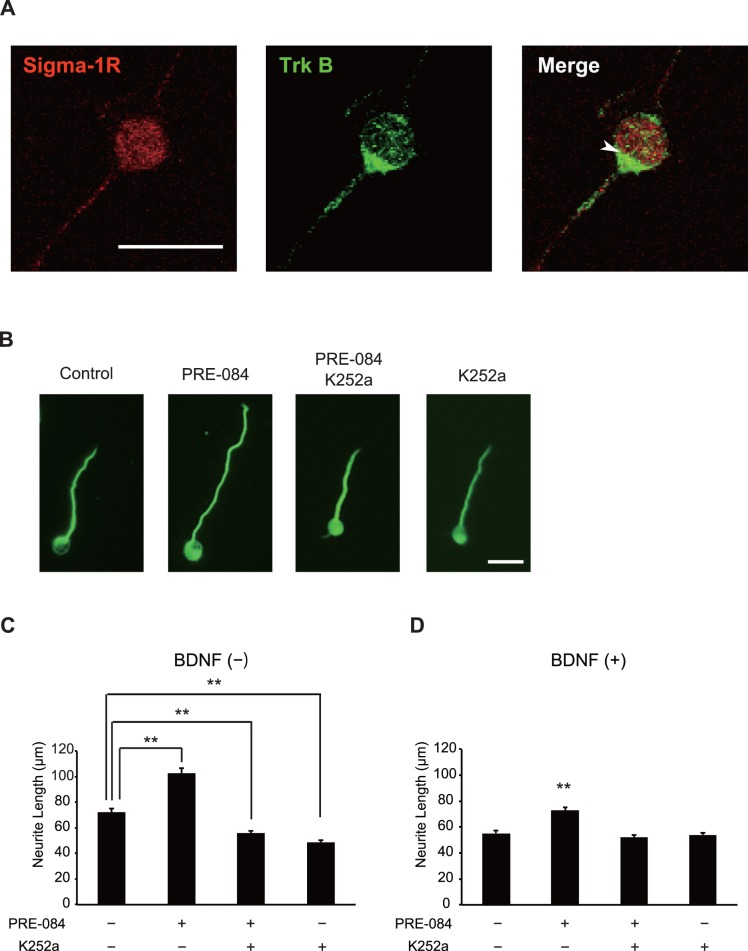
TrkB is required for the neurite elongation by PRE-084. (A) CGNs immunostained for Sig-1R (red) and TrkB (green). Scale Bar: 10 µm. (B and C) CGNs were cultured for 24 h with or without PRE-084. As for cultures incubated with K252a, a pan-tropomyosin receptor kinase (trk) K252a was added at the same time as PRE was added to the culture. Representative images of CGNs are shown in (B). The neurite lengths were quantified from three independent experiments. The mean lengths of neurite are shown in the graph (C). Scale bar: 20 µm. (D) The same effects were observed when the cells were cultured in the presence of BDNF; n = 3, **P<0.01, Scheffe’s test.

The neurotrophin receptor TrkB is well known for its role in cell survival, proliferation, and differentiation [16]. Considering TrkB is expressed predominantly in the cerebellum among other Trk family [16], and our present work confirms Sig-1R also is expressed in the CGNs, we hypothesize that TrkB participates in the Sig-1R-mediated neurite outgrowth. The observation that TrkB is partially co-localized with Sig-1R in the soma of immunostained CGNs supports this hypothesis (Fig. 2A). To determine whether TrkB activity is necessary for Sig-1R to mediate neurite outgrowth, TrkB activity was suppressed in CGNs using the pan-Trk inhibitor K252a. CGNs with or without PRE-084 were cultured with or without 50 nM K252a for 24 h and immunostained with anti-Tuj1 antibody, and the neurite lengths were then measured. The cells treated solely with PRE-084 demonstrated an increased neurite outgrowth compared to that of the control. However, when the neurons were cultured with PRE-084 and K252a, the effect of activated Sig-1R on the neurite outgrowth was abolished (Fig. 2B and C). These results indicate that Sig-1R promotes neurite outgrowth through Trk activity.

Cells treated solely with K252a showed a decreased neurite length compared with controls (Fig. 2B). This result indicates that the Trk endogenously regulates neurite elongation in CGNs. PRE-084 treatment also enhanced neurite elongation in the presence of brain-derived neurotrophic factor (BDNF), a ligand for TrkB, and the effect of PRE-084 was also abrogated by K252a treatment (Fig. 2D). These results suggest that Sig-1R promotes neurite outgrowth through BDNF-dependent and BDNF-independent TrkB activity.

### Sig-1R Interacts with TrkB in CGNs

**Figure 3:**
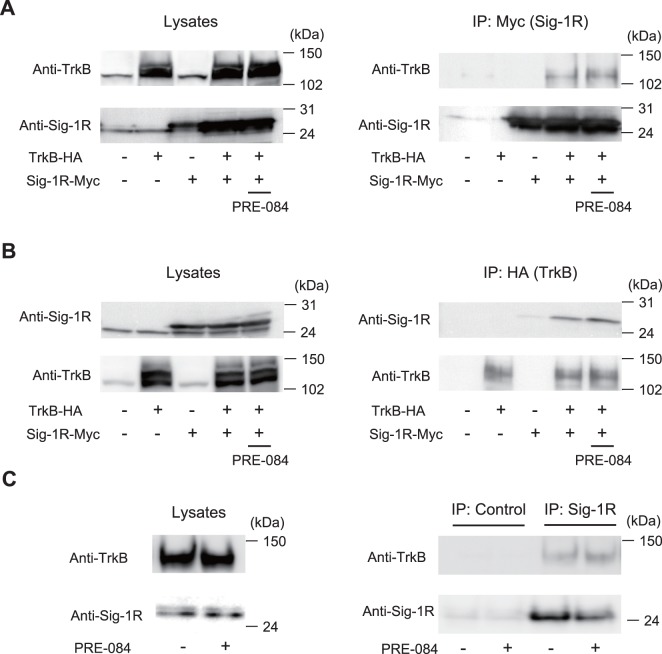
Sig-1R interacts with TrkB in CGNs. (A and B) Co-immunoprecipitation of Myc-tagged full-length sigma-1 receptor (Sig-1R-Myc) with HA-tagged full-length TrkB (HA-TrkB). HEK 293T cells were transiently transfected with the indicated plasmids and treated with or without PRE-084 (10 µM) for 1 h. Cell lysates were immunoprecipitated with the anti-Myc antibody (A) or anti-HA antibody (B). The immunoprecipitates (IP) and cell lysates (Lysates) were analyzed by immunoblotting with anti-HA and anti-Myc antibodies. (C) Association of endogenous Sig-1R with TrkB in the CGNs. The CGNs were treated with or without PRE-084 (20 µM) for 1 h. The lysates prepared from the CGNs were immunoprecipitated with anti-Sig-1R antibody followed by immunoblotting with anti-TrkB and anti-Sig-1R antibodies, respectively. The association between Sig-1R and TrkB was slightly enhanced in the PRE-084-treated cells. Control: PBS.

We then examined whether Sig-1R interacts with TrkB. HEK 293T cells were transfected with Myc-tagged full-length Sig-1R and/or hemagglutinin (HA)-tagged full-length TrkB. These cell extracts were immunoprecipitated with anti-Myc or anti-HA antibodies respectively (Fig. 3A and B). Of the Sig-1R-Myc immunoprecipitates, HA-TrkB was detected only in the Sig-1R-Myc and HA-TrkB co-transfected cells (Fig. 3A). The results were consistent when HA-TrkB was immunoprecipitated with anti-HA antibodies (Fig. 3B). These findings suggest that ectopically expressed Sig-1R interacts with TrkB in HEK 293T cells. Next, the interaction of endogenous Sig-1R and TrkB were examined in postnatal day 7 CGNs. These cells were immunoprecipitated with anti-Sig-1R antibodies and immunoblotted with TrkB antibodies. TrkB was detected in the immunoprecipitates obtained with Sig-1R antibody (Fig. 3C). These results indicate the physical interaction of endogenous Sig-1R and TrkB in CGNs.

### Up-regulation of Y515 is Necessary for Sig-1R-mediated Neurite Outgrowth

**Figure 4:**
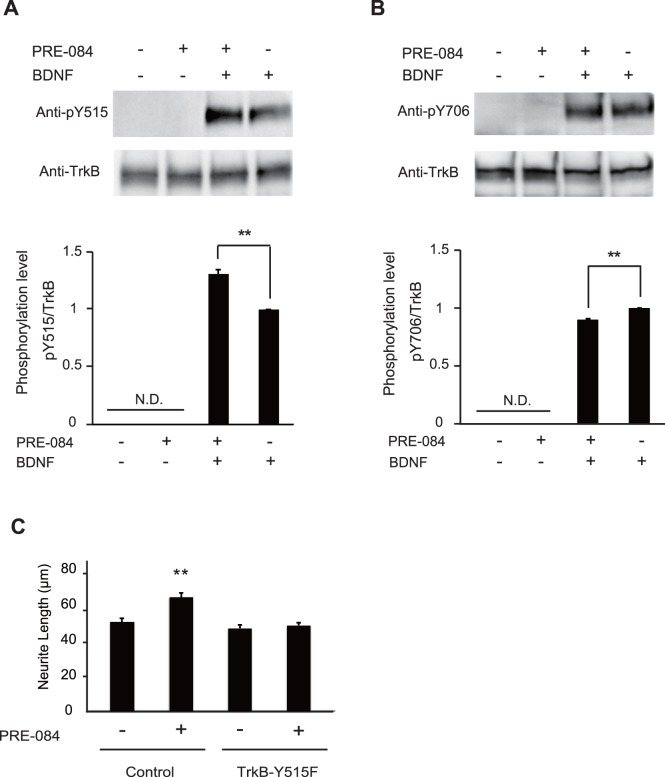
Up-regulation of Y515 is necessary for Sig-1R-mediated neurite outgrowth. (A and B) The panels show representative western blots for detection of phosphorylation of Y515 (A) or Y706 (B) of TrkB and total TrkB. The graphs demonstrate relative phosphorylation level of Y515 (A) or Y706 (B) of TrkB. The Sig-1R activation by PRE-084 significantly promoted phosphorylation of Y515 of TrkB by 30%, whereas the phosphorylation level at Y706 significantly decreased by 10%. Without BDNF, PRE-084 alone did not increase TrkB phosphorylation. ND: not determined, (A) n = 5, (B) n = 3, **P<0.01, Scheffe’s test. (C) Dominant negative knockdown of endogenous TrkB attenuated the effects of PRE-084 on neurite growth. The neurons transfected with control vector, or TrkB-Y515F were cultured for 24 h in the presence or absence of PRE-084. n = 3, **P<0.01, Scheffe’s test.

Our results thus far indicate Sig-1R enhances neurite outgrowth through TrkB (Fig. 2A–D), and these two receptors interact in CGNs (Fig. 3A–C). We therefore attempted to find out how the interaction contributes to the promotion of neurite elongation. We hypothesize that Sig-1R stimulation enhances TrkB activation. It was reported previously that tyrosine phosphorylation of TrkB is critical for its function [17]. To examine whether Sig-1R promotes neurite outgrowth through TrkB activation, we examined the phosphorylation level of TrkB upon Sig-1R stimulation. In particular, we examined the phosphorylation levels of the three major cytoplasmic tyrosines (Y515, Y706, and Y816). In the presence of BDNF, phosphorylation levels of Y515, known as the Shc docking site, were increased by 30% upon PRE-084 stimulation, whereas phosphorylation levels of Y706, a catalytic site, decreased by 10% (Fig. 4A and B). Phosphorylation levels of Y816, the PLCγ binding site, could not be detected under our experimental conditions (data not shown). To investigate whether Sig-1R activation alone can induce TrkB phosphorylation, we examined TrkB phosphorylation levels in the absence of BDNF. CGNs were treated only with PRE-084 for 1 h, followed by western blotting with the indicated antibodies. Unexpectedly, phosphotyrosine signal was undetected without BDNF (Fig. 4A and B, 2 left lanes), but was detected in the presence of 100 ng/mL BDNF (Fig. 4A and B, 2 right lanes). Furthermore, to confirm a role of phosphorylated Y515 on TrkB in this Sig-1R-mediated neurite outgrowth, activities of Y515 was abrogated by transfecting CGNs with plasmids that carry TrkB with a substitution of phenylalanine for tyrosine at 515 (TrkB-Y515F). Eliminating tyrosine at 515 on TrkB significantly abolished the Sig-1R-induced neurite outgrowth in CGNs (Fig. 4C). These results further support our hypothesis that Sig-1R stimulation enhances TrkB activity and that promotes neurite outgrowth.

## Discussion

In the present study, we demonstrated that Sig-1R promotes neurite outgrowth through TrkB activity in CGNs. This effect is supported by two pieces of evidence. First, activated Sig-1R promotes tyrosine phosphorylation on TrkB (Fig. 4A and B). Second, TrkB inactivation by K252a as well as disruption of phosphorylation on Y515 of TrkB abrogated the neurite elongation mediated by Sig-1R (Fig. 2A–C and Fig. 4C–D). Although Sig-1R is known to promote neurite elongation, the underlying molecular mechanisms remain elusive. Our results indicate that Sig-1R modulates TrkB activity and promote neurite elongation through physical interaction (Fig. 3A–C). This study therefore provides a new perspective on Sig-1R function in the CNS.

Previous reports have described the interactions between Sig-1R and several ion channels, which include potassium channels, voltage-gated calcium channels, and acid-sensing ion channels on plasma membrane [18]–[20]. In this study, we demonstrate that Sig-1R physically interacts with TrkB in transfected HEK 293T cells, as well as in CGNs (Fig. 3A–C). Interactions between Sig-1R and TrkB were slightly enhanced by PRE-084 treatment (Fig. 3A–C). Furthermore, we found that PRE-084 treatment increased BDNF-dependent TrkB phosphorylation (Fig. 4A). Considering these results, it is intriguing to hypothesize that Sig-1R has kinase activity. The direct interactions between Sig-1R and TrkB should be examined further in future studies.

We observed that TrkB phosphorylation was not detected without BDNF application, even in the presence of 10 µM PRE-084 (Fig. 4A and B). Nevertheless, this condition was sufficient to induce neurite elongation in this study (Fig. 2B), and in the study performed by Guzman-Lenis *et al.* [11]. These observations might be explained by two distinct possibilities. First, phosphorylation levels are far too low to be detected via western blotting. This first assumption might be verified with an enzyme-linked immunosorbent assay for phosphorylated TrkB, or through the identification of an optimal PRE-084 concentration for detection of phospho-TrkB. As the drug works on Sig-1R in a dose-dependent manner [21], an optimal concentration of PRE-084 that maximizes the phosphorylation of TrkB might enable us to detect trk phosphorylation without BDNF. This might explain the observation that phosphorylation was not detected in the control cultures although endogenous TrkB activity on neurite outgrowth without BDNF was suggested by K252a (Fig. 2A–C).

The second assumption is that in the absence of BDNF, activated Sig-1R interacts with molecules downstream of Trk signaling and initiates transduction cascades without modifying Trk activity itself. This assumption might be supported by the finding that Sig-1R increases intracellular Ca^2+^ levels upon activation [22]. Altering intracellular Ca^2+^ concentration modulates cellular activities, including regulation of the transduction signaling pathway [23]. Indeed, it has been reported that Sig-1R regulates PKA- and PKC-dependent pathways [21]. Our results regarding phosphorylation levels of TrkB upon Sig-1R activation also support this assumption. PRE-084 treatment along with BDNF decreased Y706 phosphorylation levels, while it increased Y515 phosphorylation levels (Fig. 3A–B). It is well known that Y706 is the catalytic domain of TrkB, and Y515 is the Shc docking site, which mediates the Ras-dependent MAPK signaling cascade [17], [24]. Our results therefore suggest that while the catalytic activity of TrkB is downregulated, the Shc-dependent cascade may be upregulated through Sig-1R activation.

Based on the neurite lengths of the control groups cultured with or without BDNF, BDNF alone did not appear to promote neurite elongation (the actual mean lengths were 72.13 and 55.27 µm, respectively; Fig. 2B–C). This observation agrees with a previous report that demonstrated P3–4 rat CGN outgrowth both with and without BDNF [25]. Nevertheless, PRE-084 treatment with BDNF promoted neurite outgrowth (Fig. 2C) and increased the Y515 phosphorylation levels (Fig. 4A). In addition, our data suggest that by inducing a point mutation at Y515 on TrkB, Sig-1R-promoted neurite outgrowth in the absence of BDNF was completely abolished (the actual mean lengths were 70.5 µm and 53.1 µm respectively; Fig. 4C). This showed that Y515 plays an essential role in the mechanism through which TrkB activity is regulated by Sig-1R.

In conclusion, we showed that Sig-1R promotes neurite elongation via TrkB activity, regardless of the presence of BDNF. Moreover, in the presence of BDNF, Sig-1R promotes Y515 phosphorylation beyond that derived by BDNF alone. Further examining the molecular mechanisms involved, such as the phosphorylation of molecules downstream of TrkB signaling, might lead to the discovery of other Sig-1R functions.

## Materials and Methods

### Animals

All procedures were approved by the Guidelines for the Care and Use of Laboratory Animals of the Graduate School of Medicine, Osaka University. C57BL/6J mice purchased from Kiwa Animal Farm (Wakayama, Japan) or Japan SLC Inc. (Shizuoka, Japan) were used in the study.

### Reagents and Antibodies

The following reagents were used in this study: 2-(4-Morpholinethyl)1-phenylcyclohexanecarboxylate (PRE-084), a specific Sig-1R agonist; 1-[3,4-dichlorophenyl]ethyl]-4-methylpiprazine (BD1063), a Sig-1R-specific antagonist (Tocris Bioscience, Minneapolis, MN, USA), brain-derived neurotrophic factor (BDNF; Peprotech, Rocky Hill, NJ, USA), and K252a (Alomone Labs, Jerusalem, Israel) were used.

For immunoprecipitations, western blots, and immunostainings, the following primary and secondary antibodies were used: goat anti-human Sigma-1 receptor (Santa Cruz Biotechnology, Inc., Santa Cruz, CA, USA), Goat TrueBlot: horseradish peroxidase (HRP)-conjugated anti-goat immunoglobulin G (IgG) (eBioscience, San Diego, CA, USA), rabbit neuronal class III ß-tubulin polyclonal antibody (Tuj1; Covance Laboratories, Emeryville, CA, USA), donkey anti-goat Alexa 568-conjugated IgG (Invitrogen, Carlsbad, CA, USA), goat anti-rabbit Alexa 488-conjugated IgG (Invitrogen), biotinylated anti-mouse TrkB antibody (R&D Systems, Minneapolis, MN, USA), streptavidin-HRP-conjugated IgG (Roche Diagnostic, Basel, Switzerland), phospho-TrkA (Tyr 674/675)/TrkB (Tyr 706/707) rabbit monoclonal antibody (Cell Signaling Technology, Danvers, MA, USA), phospho-TrkA (Tyr 490)/TrkB (Tyr 516; Cell Signaling Technology) which detects phosphorylation of mouse Tyr 515, HRP-conjugated anti-rabbit IgG (Cell Signaling Technology), HRP-conjugated anti-mouse IgG (Cell Signaling Technology), c-Myc (9E10) (Santa Cruz), and rabbit polyclonal anti-HA tag (Abcam, Cambridge, MA, USA).

### Cell Culture

Human embryonic kidney cell line, HEK 293T cells, were cultured in DMEM (Invitrogen) and supplemented with 10% (*v/v*) FBS in humidified incubator maintained at 37°C and 5% CO_2_ atmosphere. Primary dissociated cultures of CGNs were prepared from 7- to 9-day-old C57BL/6J mice using a previously described protocol [26]. Briefly, CGNs were dissected out, minced into small pieces on ice, and then collected in ice-cold 0.01 M phosphate-buffered saline (PBS). The cells were then incubated with 0.25% trypsin (Gibco/Invitrogen, Paisley, UK) and 500 µg/mL DNase1 (Takara, Shiga, Japan) at 37°C for 15 min. To terminate the trypsinization, Dulbecco’s modified Eagle’s medium/Nutrient Mixture F-12 (DMEM/F12) containing 10% (*v*/*v*) fetal bovine serum (FBS) was added and gently mixed by tube inversion. The cells were centrifuged at 1000 rpm for 4 min at 4°C. The medium was replaced with fresh DMEM/F12 supplemented with 10% FBS, and cells were resuspended, and further dissociated by gentle pipetting. Cell debris and undissociated neurons were omitted by passing the sample through 70 µm pore filters. The filtered cells were centrifuged at 1000 rpm for 3 min at 4°C, and the medium was aspirated. The cells were then washed with fresh DMEM/F12 containing 10% FBS and again centrifuged. After the medium was aspirated, the cells were resuspended in DMEM/F12 supplied with B27 (Invitrogen). The number of living neurons (trypan blue negative) was counted; dead cells (trypan blue positive) were excluded from counting.

For the neurite outgrowth assay, neurons were plated at a density of 6×10^4^ cells/φ35-mm in a culture dish precoated with poly-l-lysine (PLL) (2.5×10^6^ cells/precoated φ35-mm dishes for western blotting and 7–8×10^6^ cells/precoated φ35-mm dishes for immunoprecipitation). The resulting cells were then incubated in a chamber as described above.

### Nucleofection

CGNs were isolated and dissociated from P7 to P9 mice as described above. The cells were washed and resuspended in room temperature Mouse Neuron Nucleofector Solution (Amaxa; Lonza Cologne AG, Cologne, Germany) at a final concentration of 5×10^6^ cells per 100 µL. The cell–nucleofector solution complex (100 µL) and the mouse TrkB-Y515F plasmid or empty vector (7 µg) were then gently mixed and transferred into a cuvette, followed by nucleofection using the nucleofector program O–05. Immediately after electroporation, the cells were mixed with 500 µL of pre-warmed DMEM/F12 containing 10% FBS, and the cell suspension was then transferred onto poly-L-lysine-coated dishes. The cells were placed in an incubator for 3 h, after that, the medium was replaced with fresh DMEM/F12 containing B27, and the cells were incubated for an additional 96 h. Cells were then collected and re-plated onto poly-L-lysine-coated dishes for neurite outgrowth assay.

### Immunocytochemistry

Sig-1R expression was examined in CGNs, by preparing primary cultures and incubating them for 24 h in the incubation chamber. The cells were fixed with 4% (*w*/*v*) paraformaldehyde (PFA) for 40 min at room temperature (RT) and rinsed thoroughly three times with PBS. The cells were then permeabilized and blocked with 5% bovine serum albumin (BSA) in PBS containing 0.1% Triton-X100 for 30 min at RT. The cells were subsequently immunostained with indicated primary antibodies in blocking solution at 4°C overnight, followed by a 90-min incubation with Alexa 568-conjugated IgG (1∶500) and Alexa 488-conjugated IgG (1∶500), and a 10-min incubation with 4′, 6′-diamidino-2-phenylindole (DAPI, 1 µg/mL in MilliQ; Millipore, Billerica, MA, USA) at room temperature. The following antibodies were used for immunocytochemistry: anti-Sig-1R (1∶100), anti-TrkB (1∶50), and anti-Tuj1 (1∶1000) antibodies. To verify nonspecific antibody bindings, cell cultures were prepared that were not treated with sigma receptor primary antibody, but with Alexa 568-conjugated IgG (1∶500) and DAPI. 5% BSA in PBS/0.1% Triton-X100 was used as a vehicle. The staining was observed, and pictures were taken using a BX51 upright microscope, U-RFL-T power supply unit, and DP70 Digital Camera System (Olympus, Tokyo, Japan).

### Neurite Outgrowth

To investigate the effect of PRE-084 and BD1063 on neurite outgrowth, the dissociated CGNs were treated with 10 µM PRE-084 and/or 10 µM BD1063 upon plating and allowed to incubate for 24 h in the chamber. To investigate the effect of K252a treatment on neurite outgrowth upon Sig-1R activation, the cells were exposed to 50 nM K252a and/or 10 µM PRE-084 at the same time and cultured for 24 h. The cells were then fixed and blocked in the same way as described in the Immunocytochemistry section. The cells were then immunostained with anti-Tuj1 overnight at 4°C, followed by a 90-min incubation with Alexa 488-conjugated IgG and a 10-min incubation with DAPI at RT. The lengths of the longest cell neurites were measured using the ImageJ software (available as a public domain software through the National Institutes of Health, MD, USA). Cells with neurites shorter than the diameter of its soma were excluded from the analysis.

### Plasmid Construction and Transfection

To generate Myc tagged human Sig-1R expression vector, the sequence encoding Myc tag was added to C-terminal of Sig-1R human cDNA clone (SC111748; OriGene, Rockville, MD, USA) by PCR, and the cDNA was cloned into pcDNA 3.1 (Invitrogen). The construct was verified by DNA sequencing. Hemagglutinin-tagged TrkB in pcDNA 3.1 was a generous gift from Dr. Barde YA [27]. HEK 293T cells were transfected with these plasmids using Lipofectamine 2000 (Invitrogen) according to manufacturer’s instructions. The cells were lysed 48 h after the transfection, and used for immunoprecipitation. pcDNA3.1 plasmid vector that contains TrkB–Y515F has been produced and used in our previous report [28].

### Co-immunoprecipitation

Cells were washed with ice-cold PBS and lysed on ice in lysis buffer (50 mM Tris-HCl (pH 8.0), 150 mM NaCl, 1% NP-40, 1 mM EDTA, 10% glycerol) containing a protease inhibitor cocktail (Roche Diagnostics K.K., Tokyo, Japan), incubated for 20 min at 4°C with rotation, followed by centrifugation at 4°C with 15000 rpm for 10 min. The supernatants were incubated with the indicated antibodies (2 µg/sample) for 2 h at 4°C rotated. The immune complexes were collected with protein A or protein G-Sepharose (GE Healthcare, Chalfont St Giles, England) for 1 h at 4°C. After washing the beads four times with the lysis buffer, the proteins were eluted by boiling in 40 µL of 2× sample buffer (250 mM Tris-HCl (pH 6.8), 5% (*v/v*) SDS, 0.05% (*w/v*) bromophenol blue, 40% (*v/v*) glycerol, and 25% (*v/v*) ß-mercaptoethanol) for 5 min, and subjected to sodium dodecyl sulphate polyacrylamide gel electrophoresis (SDS-PAGE), followed by western blotting. Where indicated, the cells were treated with 10 µM PRE-084.

### Western Blot Analysis

To determine the level of BDNF-induced TrkB phosphorylation upon Sig-1R activation, dissociated CGNs were cultured for 48 h before being harvested. At 47 h, 10 µM PRE-084 was added to the designated dishes and incubated for another hour. After the incubation, 100 ng/mL BDNF was added to dishes and incubated for 5 min. The cells were then harvested. For the analysis of phosphorylation level, samples were obtained in the same procedures described above; only the lysis buffer also contains 10 mM NaF, 1 mM Na_3_VO_4_ as phosphatase inhibitors. As for immunoprecipitation samples, the same harvesting method was performed except PRE-084 was added to indicate samples an hour prior to the harvesting.

Samples were boiled in sample buffer for 5 min. Proteins were separated on a 7.5% or 15% SDS polyacrylamide gel or purchased 4–20% gradient gel (Cosmo Bio, Tokyo, Japan). These proteins were transferred to a methanol-immersed polyvinylidene difluoride membrane (Millipore). The membrane with proteins was first blocked with 5% skim milk (*w*/*v*) in Tris-buffered saline containing 0.1% (*v*/*v*) Tween 20 (TBS–T) at RT for 1 h and then incubated with primary antibody (anti-c-Myc (9E10), 1∶1000; anti-HA, 1∶1000; anti-human sigma-1 receptor antibody, 1∶200; biotinylated anti-mouse TrkB antibody, 1∶2500; anti-phospho-TrkA [Y490]/TrkB [Y516] antibody, 1∶1000; anti-phospho-TrkA [Tyr 674/675]/TrkB; Tyr 706/707 antibody, 1∶1000) diluted in 1% skim milk in TBS–T at 4°C overnight rocking. After washing the membranes four times with TBS–T, each membrane was incubated with the corresponding secondary antibody (HRP-conjugated anti-rabbit IgG antibody, 1∶5000; HRP-conjugated anti-mouse IgG antibody, 1∶5000; goat TrueBlot, 1∶1000; HRP-conjugated streptavidin, 1∶2500) diluted in 1% skim milk in TBS–T at RT for 90 min. The probed proteins on the membrane were detected using the ECL Plus reagent or ECL Advance reagents (GE Healthcare Life Sciences, Giles, UK) and visualized by LAS−3000 image system (Fuji Film, Tokyo, Japan). The signals were quantified with MultiGauge (Fuji Film).

### Statistical Analysis

All the data in this study are presented as mean ± SEM of at least three independent experiments. Statistical significances were evaluated using the Scheffe’s test. *P*-values <0.01 were considered significant.
